# Pregnancy outcomes as related to in utero exposure to air pollution and greenness: The Life-GAP Project

**DOI:** 10.1097/EE9.0000000000000318

**Published:** 2024-06-21

**Authors:** Robin M. Sinsamala, Ane Johannessen, Randi J. Bertelsen, Simone Accordini, Jørgen Brandt, Lise M. Frohn, Camilla Geels, Thorarinn Gislason, Mathias Holm, Christer Janson, Iana Markevych, Hans Orru, Francisco Gómez Real, Torben Sigsgaard, Svein M. Skulstad, Cecilie Svanes, Alessandro Marcon

**Affiliations:** a Centre for International Health, Department of Global Public Health and Primary Care, University of Bergen, Bergen, Norway; b Department of Global Public Health and Primary Care, University of Bergen, Bergen, Norway; c Department of Clinical Science, University of Bergen, Bergen, Norway; d Unit of Epidemiology and Medical Statistics, Department of Diagnostics and Public Health, University of Verona, Verona, Italy; e Department of Environmental Science, Aarhus University, Frederiks-borgvej, Roskilde, Denmark; f Faculty of Medicine, University of Iceland, Reykjavik, Iceland; g Department of Sleep, Landspitali University Hospital; h Occupational and Environmental Medicine, School of Public Health and Community Medicine, Institute of Medicine Sahlgrenska Academy, University of Gothenburg, Gothenburg, Sweden; i Department of Medical Sciences, Respiratory, Allergy & Sleep Research, Uppsala University, Uppsala Sweden; j Institute of Psychology, Jagiellonian University, Krakow, Poland; k Health and Quality of Life in a Green and Sustainable Environment, SRIPD-MUP, Medical University of Plovdiv, Plovdiv, Bulgaria; l Institute of Family Medicine and Public Health, University of Tartu, Tartu, Estonia; m Department of Obstetrics and Gynecology, Haukeland University Hospital, Bergen, Norway; n Department of Public Health, Environment Occupation and Health, Danish Ramazzini Centre, Aarhus University, Aarhus, Denmark; o Department of Occupational Medicine, Haukeland University Hospital, Bergen, Norway

**Keywords:** Air pollution, Greenness, Birth weight, Preterm birth, Pregnancy

## Abstract

**Background:**

Lower birth weight and preterm birth may increase the risk of adverse health outcomes later in life. We examined whether maternal exposure to air pollution and greenness during pregnancy is associated with offspring birth weight and preterm birth.

**Methods:**

We analyzed data on 4286 singleton births from 2358 mothers from Respiratory Health in Northern Europe, a prospective questionnaire-based cohort study (1990–2010). Mixed-effects regression models with random intercepts for mothers and centers were used to estimate the association of exposures to particulate matter (PM_2.5_ and PM_10_), nitrogen dioxide (NO_2_), ozone (O_3_), black carbon (BC), and greenness (Normalized Difference Vegetation Index in 300m-buffers [NDVI_300m_]) with birth outcomes, adjusting for potential confounders.

**Results:**

Median (interquartile range [IQR]) exposures to PM_2.5_, PM_10_, NO_2_, O_3_, BC, and NDVI_300m_ during pregnancy were 8.4(5.0) µg/m^3^, 14.4(8.3) µg/m^3^, 14.0(11.0) µg/m^3^, 54.7(10.2) µg/m^3^, 0.47(0.41) µg/m^3^, and 0.31(0.20), respectively. IQR increases in air pollution exposures during pregnancy were associated with decreased birth weight and the strongest association was seen for PM_2.5_ (−49g; 95% confidence interval [CI] = −83, −16). However, O_3_ showed an opposite association. IQR increase in NDVI_300m_ was associated with an increase in birth weight of 25 g (95% CI = 7, 44). Preterm birth was not associated with the exposures.

**Conclusion:**

Increased greenness and decreased air pollution may contribute to healthier pregnancies and improve overall health in the next generation. This emphasizes the need to adopt policies that target the reduction of air pollution emissions and exposure of the population.

**What this study adds**

This study documented the association between in utero exposure to air pollution and the risk of lower birth weight in Northern European countries, where air pollution concentrations are relatively low. It also strengthens the knowledge base related to the beneficial effect of greenness (Normalized Difference Vegetation Index) exposure with birth weight. The associations estimated for Normalized Difference Vegetation Index exposure were independent from concomitant air pollution exposures, as illustrated by mutually adjusted models. Sensitivity and secondary analyses validated the robustness of the observed associations. Further analysis on exposure to PM and NO_2_ above the WHO air quality guidelines levels and birth weight highlighted the need to adhere to WHO recommendations.

## Introduction

Adverse birth outcomes that are markers of intrauterine growth retardation, such as low birth weight, preterm birth, and small for gestational age, have been linked to air pollution exposure during pregnancy.^1^ Low birth weight, (LBW), high birth weight (HBW), and preterm birth (PTB) are all associated with other detrimental neonatal outcomes and severe long-term health effects extending into adulthood.^2–4^ Plausible biological mechanisms, such as oxidative stress, inflammation, mitochondrial and nuclear DNA methylation, and endocrine disruption, have been hypothesized to explain some of the negative pregnancy outcomes associated with exposure to air pollution.^5^ Recent evidence also suggests that preconception exposure in the parental generation may influence the health outcomes of the offspring through epigenetic mechanisms. It is proposed that parental exposure to adverse environmental conditions could alter the germ cell epigenome structure, which may then be transmitted to the offspring and impact different health outcomes.^6,7^

Emerging evidence from several studies suggests that greenness may mitigate the harmful health effects caused by air pollution.^8,9^ Exposure to greenness has shown a positive impact on health, including pregnancy outcomes; however, some of the findings are inconclusive.^10^ Although the mechanisms are not fully understood, certain pathways have been proposed to elucidate how greenness may be beneficial for improved pregnancy outcomes, including promoting physical activity, reducing psychophysiological stress, reducing environmental exposures such as ambient air pollution and high temperature, and improving mental health by promoting interpersonal contacts and community belonging.^11,12^

There is substantial evidence supporting the adverse impact of prenatal air pollution exposure with several adverse birth outcomes, although heterogeneity between studies exists.^13–16^ For instance, consistent associations have been shown between particulate matter (PM) exposure during pregnancy and LBW, while there are less consistent associations of PM exposure with PTB or macrosomia.^1^

Most evidence on the effects of in utero and prenatal exposures derives from studies conducted in settings where air pollution concentrations largely exceed the air quality guideline (AQG) levels recommended by the WHO.^17,18^ However, it is imperative to conduct research also in low air pollution settings to gain a comprehensive understanding of the effects of exposures across a spectrum of air pollution concentrations. This is essential in striving toward air quality levels that truly safeguard the most vulnerable individuals.^19^ Using data from the Respiratory Health in Northern Europe (RHINE) study, we aimed to investigate whether maternal exposure to air pollution and greenness during pregnancy are associated with offspring birth weight and PTB. As a secondary aim, we also assessed the relationship between maternal exposure to air pollution and greenness before conception with offspring birth weight.

## Methods

### Study design and population

**Figure 1.**
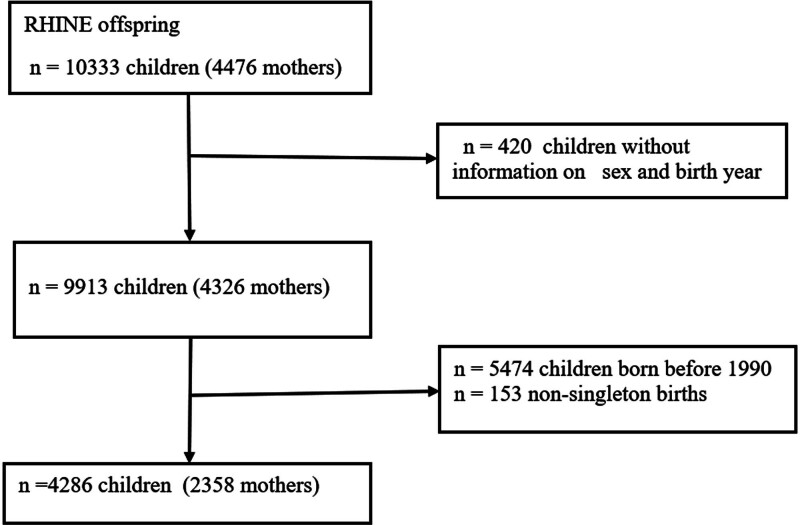
Flowchart of the Respiratory Health in Northern Europe (RHINE) study participants.

RHINE is a prospective questionnaire-based cohort study on a general population aged 20–44 years at baseline that started in 1990 (RHINE 1) with follow-ups every 10 years (ca. 2000 and 2010). It includes seven study centers: Bergen (Norway); Gothenburg, Umea, and Uppsala (Sweden); Aarhus (Denmark); Reykjavik (Iceland); and Tartu (Estonia). The study received approval from the ethical review boards in all centers, according to national legislation, and before participation, written informed consent was obtained. We analyzed birth outcomes of singleton children born to female participants in RHINE after 1990. This year was chosen based on the availability of modeled residential air pollution exposures. Multiple births were excluded a priori as the overall in utero growth pattern is slower for multiple fetuses compared with singletons^20^ (Figure 1).

### Birth outcomes

We examined the following birth outcomes: birth weight (continuous [grams, g]), and the binary outcomes LBW (birth weight <2500 g), HBW (birth weight >4500 g); and PTB (gestation age <37 weeks).^21^ Data on pregnancy outcomes were obtained through maternal reporting in the second follow-up (RHINE III) for all study centers (except for birth weight data for Reykjavik, which was retrieved from the Icelandic birth registry), along with detailed information on lifestyle factors and comorbidities. The questionnaires concerning pregnancy-related outcomes were validated in a study performed in the Bergen study centre in Norway; agreement for LBW was good (Cohen’s κ = 0.73–85), while PTB showed moderate agreement (Cohen’s κ = 0.60).^22^

### Exposure assessment

We assigned in utero exposures to air pollutants and greenness to each of the children as follows. First, we assigned annual average exposures to the addresses reported by the mothers at RHINE I, II, and III using the methods described in sections 2.4 and 2.5, respectively. Second, we identified the address where the mother lived during the year of pregnancy, which was defined as the year preceding birth year since the exact birth dates were not available. For this purpose, we reconstructed maternal residential histories backward based on information on how long mothers had been living at the addresses reported in RHINE III. For mothers who had moved during the RHINE follow-up period (1990–2010), we used residential addresses and times reported in RHINE II, and residential addresses reported at RHINE I. This resulted in three groups of children assigned to addresses reported in RHINE I, II, and III. Finally, we linked each of these groups to the relevant exposure estimates.

### Air pollution exposure

Annual average air pollution concentrations (micrograms per cubic meter, µg/m^3^) used in this study were obtained from the NordicWelfAir inventory (https://projects.au.dk/nordicwelfair) for all the study centers except Tartu, which was assigned locally. We obtained data for PM with an aerodynamic diameter of ≤2.5 µm and ≤10 µm (PM_2.5_ and PM_10_), nitrogen dioxide (NO_2_), ozone (O_3_), and black carbon (BC). Concentrations were calculated based on a combined multiscale air pollution modeling system which includes long-range transported air pollution (regional sources), meteorological variations, and local source contribution at high temporal and spatial resolutions.^23^ Two models were combined: the Danish Eulerian Hemispheric Model (DEHM)^24^ and the Urban Background Model (UBM).^25^ The DEHM is a 3-dimensional chemistry-transport model that covers the Northern Hemisphere with a horizontal resolution of 150 km × 150 km. It includes nested domains over Europe with a resolution of 50 km × 50 km and Northern Europe (16.67 km × 16.67 km resolution). The model has 29 vertical layers and covers the lowest approximately 15 km of the atmosphere. It calculates the transport, dispersion, deposition, and chemistry of both anthropogenic and natural emissions. The meteorological data needed for the DEHM (and UBM) model(s) were available from the Weather Research and Forecasting model.^26^ The DEHM output contains concentration distributions of pollutants that originate from anthropogenic or natural emissions and include primary emitted particles and gases and their chemistry.^24^ Integrated with DEHM is the UBM, which calculates the transport and dispersion of primary emitted air pollutants from all local sources, such as traffic and residential heating, industry, agriculture, power plants, and so on, and calculates air pollution concentrations, including chemistry of NO, NO_2,_ and O_3_, on a 1 km × 1 km resolution up to 25 km from all grid points, covering the Nordic countries.^25,27,28^ Both DEHM and UBM are driven with data from a meteorological model (Weather Research and Forecasting model). The DEHM/UBM air pollution concentrations were evaluated against measurements with correlation coefficient ranging from 0.43 to 0.96 for PM_2.5_, 0.72 to 0.89 for NO_2_, and 0.60 to 0.73 for O_3_ in Denmark, Finland, Norway, and Sweden.^25^ For Tartu, the data of BC and O_3_ were not available, but PM_2.5_, PM_10_, and NO_2_ were modeled using the Eulerian air quality dispersion model with the resolution of 1 km × 1 km across Estonia that is part of the Airviro Air Quality Management System (Airviro 2011 [http://smhi.se]). Airviro is a widely used web-based air pollution data management tool that uses data on air pollution emission, ground-level concentrations, and meteorological variables to perform air pollution dispersion modeling and mapping. This has been used in several epidemiological studies.^29,30^ Detailed description of the model is found in the Airviro User Documentation.^31^

### Greenness exposure

Exposure to greenness was assessed using the Normalized Difference Vegetation Index (NDVI)^32^ as a proxy for the overall greenness level, which ranges from −1 to +1. Values closer to +1 indicate greener and denser vegetation. Values close to zero indicate arid areas of rocks, sand, or snow, while negative values usually denote water.^33^ For all the study centers, NDVI rasters were calculated, and NDVI values were assigned to the residential addresses of the participants, using Landsat 4–5 Thematic Mapper TM satellite images retrieved during the most vegetation-rich months (around July–August), closest in time to each RHINE survey. We used mean NDVI values (not restricting to positive values) calculated in circular buffers of 300 m around the participants residential address (NDVI_300m_) in accordance with WHO recommendations.^34^

### Covariates

Data were obtained from maternal reporting at RHINE III. To identify potential covariates to consider for the analyses, all relevant covariates associated with the exposure and/or the outcome were identified through a review of existing literature,^35,36^ and a directed acyclic graph (DAG) was made (Figure S1; http://links.lww.com/EE/A287). Among the pregnancy-specific covariates (level-1), infants’ sex (male or female) was included as a precision variable,^37^ whereas the following were considered as potential confounders: maternal age at child’s birth (continuous, years), parity (primiparous or multiparous), maternal smoking status, and pack-years before pregnancy (coded on 4 levels: light smoking [<5 pack years], moderate smoking [5–10 pack years], heavy smoking [>10 pack years] or no smoking [never smoker]). The mother-specific variables (level 2) considered as potential confounders were obesity at menarche (as an indicator of prepregnancy body composition, obese ≥5 or nonobese <5 on body silhouette chart)^38^ and educational level (low [primary and secondary] or high [college/university]). Finally, the level-1 variables gestational age (preterm [<37 weeks], term-birth [≥37–42 weeks], or post-term [>42 weeks]) and maternal comorbidities (presence of gestational diabetes, presence of hypertension + proteinuria) were considered as potential mediators of the relationship between environmental exposures and birth weight.

### Statistical analysis

Mean (standard deviation [SD]) and median (interquartile range [IQR]) were used to describe the variables. Pearson’s correlation was used to examine the correlation among exposure variables.

#### Pregnancy exposure analysis

Three levels (level-1: offspring; level 2: mother; and level 3: center) linear (for birth weight g) and logistic regression models (for LBW, HBW, and PTB) with random-intercept terms for mothers and centers were used to examine the association with air pollutants and greenness exposures during pregnancy. All the children born at >42 weeks were excluded from the analysis of PTB (reference category: term-birth). Estimates were presented as changes in birth weight in g with 95% confidence intervals (CIs) and odds ratio (OR) with 95% CIs for the binary outcomes, computed per IQR increases in PM_2.5_, PM_10_, NO_2_, O_3_, and BC, and NDVI_300m_. Data were missing for maternal education level (0.5%), gestational age (1%), obesity (1.8%), birth weight g (5%), and maternal smoking status before pregnancy (7%). Missing values were handled through a list-wise deletion approach. We considered three different adjustment sets. The first model only considered the hierarchical data structure (offspring, mothers, and centers). The second model (main model) included infant sex, maternal age at birth, parity, maternal smoking status before pregnancy, maternal obesity at menarche, and maternal education level. The third model (“co-exposure” model) further included greenness in the analysis of air pollution associations with birth outcomes; for greenness, each air pollutant (PM_2.5_, PM_10_, NO_2_, O_3_, and BC) was included separately as an additional covariate. We did not include a model with all co-exposures because of the risk of multicollinearity due to the high correlation across air pollution exposures (Figure S4; http://links.lww.com/EE/A287).

Due to the low event rate of the binary outcomes (LBW, HBW, and PTB), subsequent analyses only focused on birth weight.

Further, we investigated the potential for effect modification by child sex (male, female) since studies have shown sex differences in the susceptibility of fetuses to oxidative stress, which is one of the mechanisms through which air pollution exposure may adversely affect birth outcomes.^39,40^ For this purpose, we used stratified analyses and tested child sex × air pollution (or greenness) interaction terms in joint models.

As part of sensitivity analyses, we reanalyzed the birth weight model by excluding Tartu since the exposure data were derived from a different model system. We also explored the influence of potential mediators (comorbidity and gestational age) in the model by including each of them in a separate model for birth weight. While the main analysis focused on the total effect of exposures, this analysis estimated the effect of the exposure through pathways that did not pass through the mediators (controlled direct effects).^41^

In line with the WHO recommendations, we also analyzed the association of air pollution above vs. below the AQG levels (PM_2.5_, PM_10_, and NO_2_ with cutoff at 5 µg/m^3^, 15 µg/m^3^, and 10 µg/m^3^, respectively)^17^ with birth weight, and the results were presented as change in birth weight g with 95% CIs.

#### Secondary analysis (preconception exposure)

We further explored the effect of preconception exposures on birth weight in the subsample of children whose maternal addresses were assigned at RHINE II or III in the main analysis. In this subsample, we assigned exposures using maternal residential addresses reported in 1990 (RHINE I) as a proxy of preconceptional exposures. Therefore, the subgroup of children who were assigned exposures during pregnancy based on RHINE I maternal addresses were excluded from this analysis because no previous residential addresses were available. We used the same statistical model and included the same covariates considered for the main model. In this subsample of children, we also repeated the main analysis based on pregnancy exposures for the sake of comparison between the effects of preconception and pregnancy exposures.

All statistical analyses were performed using Stata 17 (StataCorp, College Station, TX, USA).

## Results

A total of 4286 children born to 2358 mothers were included in the study. As presented in Table 1, the mean maternal age at birth was 31.7 years, and most of the children were born to mothers who did not smoke before pregnancy (60%), were multiparous (79%), and had attained higher education (64%). Approximately 8% were obese at menarche, and 10% had at least one comorbidity during pregnancy. The median (Q1–Q3) pregnancy years was 1998 (1991–1999). Children had a median (Q1–Q3) birth weight of 3590 (3200–3900) g; birth weight was similar across centers, except for Reykjavik, which had a slightly higher median birth weight (Figure S2; http://links.lww.com/EE/A287). The prevalence of LBW, HBW, and PTB was 3%, 4%, and 8%, respectively (Table S1; http://links.lww.com/EE/A287).

**Table 1.** Characteristics of the study participants (n = 4286 children)

Characteristics	n (%)
Center	
Aarhus	948 (22.1)
Bergen	751 (17.5)
Gothenburg	483 (11.3)
Reykjavik	494 (11.5)
Tartu	454 (10.6)
Umea	535 (12.5)
Uppsala	621 (14.5)
Maternal age in years, mean (SD)	31.7 (4.9)
Maternal smoking status before pregnancy^a^	
Light smokers (<5 pack years)	708 (17.8)
Moderate smokers (5–10 pack years)	489 (12.3)
Heavy smokers (>10 pack years)	415 (10.4)
Never smokers	2374 (59.6)
Parity	
Primiparous	917 (21.4)
Multiparous	3369 (78.6)
Maternal education level^a^	
College/University	2719 (63.7)
Primary/Secondary	1545 (36.2)
Maternal body silhouette at menarche^a^^,^^b^	
Obese	354 (8.4)
Nonobese	3853 (91.6)
Maternal comorbidity^c^	
Yes	406 (9.5)
No	3880 (90.5)
Child’s female sex	2108 (49.2)
Birth weight^a^, grams	
Mean (SD)	3571 (576)
Median (5th–95th percentile)	3590 (2700–4500)
Birth weight categories	
LBW (<2500 g)	120 (3.0)
Normal birth weight (≥2500–4500, g)	3777 (92.6)
HBW (>4500 g)	180 (4.4)
Gestational age^a^	
PTB (<37 weeks)	336 (8.0)
Term-birth (≥37–42 weeks)	3294 (78.0)
Post-term-birth (>42 weeks)	593 (14.0)

a Missing values: maternal smoking status before pregnancy (n = 300), maternal education level (n = 22), maternal body silhouette at menarche (n = 79), birth weight (n = 210) and gestational age (n = 63).

b Defined as obese for ≥5 score and nonobese for <5 score on body silhouette chart.

c At least one among pregnancy diabetes, hypertension, and proteinuria.

HBW, high birth weight; LBW, low birth weight; PTB, preterm birth; SD, standard deviation.

The distribution of air pollution and greenness exposure during pregnancy by the center are reported in Table S2; http://links.lww.com/EE/A287 and Figure S3; http://links.lww.com/EE/A287. Overall, median (IQR) concentration for PM_2.5_, PM_10_, NO_2_, O_3_, and BC were 8.4(5.0) µg/m^3^, 14.4(8.3) µg/m^3^, 14.0(11.0) µg/m^3^, 54.7(10.2) µg/m^3^ and 0.47(0.41) µg/m^3^, respectively. The median (IQR) for NDVI_300m_ was 0.31(0.20).

We observed strong positive correlations between PM_2.5_, PM_10_, NO_2_, and BC, with Pearson’s coefficients ranging between 0.69 and 0.88 (Figure S4; http://links.lww.com/EE/A287). These pollutants were negatively correlated with O_3_ and NDVI (Pearson’s coefficients between −0.44 and −0.80). NDVI was weakly positively correlated with O_3_ (Pearson’s coefficient of 0.38).

**Figure 2.**
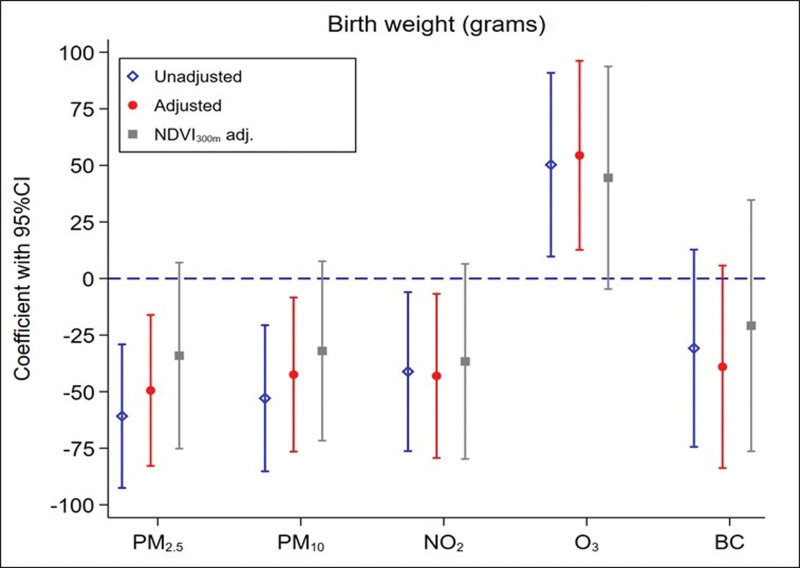
Estimated associations between residential exposures to air pollutants during pregnancy and birth weight in grams. Associations are expressed per IQR differences in air pollutants. Unadjusted, hierarchical data structure: mothers and centers. Adjusted, for child’s sex, and maternal characteristics: age, smoking status, parity, obesity, and education level. NDVI_300m_ adj., Further adjusted for greenness exposure. BC indicates black carbon; CI, confidence interval; NDVI, Normalized Difference Vegetation Index; NO_2_, nitrogen dioxide; O_3_, ozone; OR, odds ratio; PM, particulate matter.

Higher exposures to PM_2.5_, PM_10_, and NO_2_ during pregnancy were associated with lower birth weight, with estimated associations of −49 g (95% CI = −83, −16), −42 g (95% CI = −76, −8) and −43 g (95% CI = −79, 7) per IQR difference in exposures, respectively (Figure 2). Associations remained consistent across the different adjustment sets. Considering the co-exposure models (where NDVI was included), all the associations were attenuated, and the strongest and most stable association estimate was seen when adjusting for NO_2_ (−36g, 95%CI: −80, 7). We also found a positive association of O_3_ with birth weight in both unadjusted and adjusted models.

**Figure 3.**
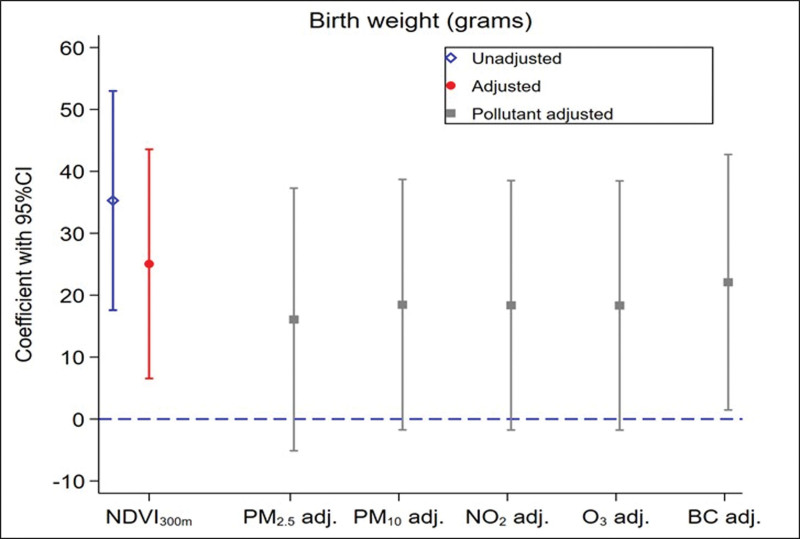
Estimated associations between residential exposure to NDVI_300m_ during pregnancy and birth weight in grams. Associations are expressed per IQR differences in NDVI_300m_. Unadjusted, hierarchical data structure: mothers and centers. Adjusted, Adjusted for child’s sex, and maternal characteristics: age, smoking status, parity, obesity, and education level. Pollutant adjusted: Further adjusted for air pollutant exposures. BC indicates black carbon; CI, confidence interval; NDVI, Normalized Difference Vegetation Index; NO_2_, nitrogen dioxide; O_3_, ozone; OR, odds ratio; PM, particulate matter.

Figure 3 shows associations of greenness exposure with birth weight. An IQR increase in NDVI_300m_ was associated with a 25 g (95% CI = 7, 44) higher birth weight in the analysis adjusted for children’s and mothers’ characteristics, which ranged from 16 g (95% CI = −5, 37) to 22 g (95% CI = 1, 43) higher birth weight when adjusting for PM_2.5_ and BC, respectively.

Table 2 shows associations of air pollution and greenness with LBW, HBW, and PTB. Overall, the results were inconclusive with LBW and HBW, but we observed a tendency of increased risk of LBW associated with an IQR increase in PM_2.5_, PM_10_, NO_2,_ and O_3_, although with wide CI. Wide CIs were also observed for PTB. All pollutant exposures were negatively (protective) associated with PTB (particularly BC), while O_3_ showed a positive (detrimental) association. No association was seen for greenness.

**Table 2.** Estimated associations of residential exposures to air pollutants and greenness (NDVI_300m_) during pregnancy with LBW, HBW, and PTB

	LBW^a^ OR (95%, CI)	HBW^a^ OR (95%, CI)	PTB OR (95%, CI)
Air pollution			
PM_2.5_ Unadjusted	1.11 (0.81, 1.53)	0.73 (0.48, 1.11)	0.82 (0.59, 1.14)
Adjusted^b^	1.14 (0.86, 1.49)	0.76 (0.48, 1.20)	0.83 (0.60, 1.15)
NDVI_300m_ adjusted^c^	1.06 (0.76, 1.46)	0.84 (0.49, 1.46)	0.87 (0.61, 1.23)
PM_10_ Unadjusted	1.22 (0.91, 1.64)	0.76 (0.49, 1.16)	0.90 (0.65, 1.23)
Adjusted^b^	1.24 (0.93, 1.65)	0.77 (0.48, 1.22)	0.92 (0.67, 1.25)
NDVI_300m_ adjusted^c^	1.19 (0.87, 1.63)	0.83 (0.49, 1.40)	0.96 (0.67, 1.34)
NO_2_ Unadjusted	1.05 (0.75, 1.49)	0.79 (0.49, 1.28)	0.76 (0.55, 1.06)
Adjusted^b^	1.14 (0.83, 1.56)	0.78 (0.48, 1.25)	0.85 (0.61, 1.16)
NDVI_300m_ adjusted^c^	1.07 (0.75, 1.53)	0.87 (0.50, 1.50)	0.98 (0.81, 1.19)
O_3_ Unadjusted	1.09 (0.70, 1.70)	1.42 (0.89, 2.25)	1.29 (0.89, 1.87)
Adjusted^b^	1.02 (0.71, 1.47)	1.52 (0.93, 2.50)	1.14 (0.79, 1.65)
NDVI_300m_ adjusted^c^	1.14 (0.76, 1.72)	1.49 (0.85, 2.62)	1.09 (0.74, 1.62)
BC Unadjusted	0.90 (0.60, 1.36)	0.82 (0.49, 1.37)	0.62 (0.42, 0.90)
Adjusted^b^	0.97 (0.68, 1.39)	0.75 (0.43, 1.29)	0.69 (0.48, 0.99)
NDVI_300m_ adjusted^c^	0.88 (0.59, 1.31)	0.75 (0.39, 1.47)	0.74 (0.50, 1.11)
Greenness			
NDVI_300m_ Unadjusted	0.88 (0.72, 1.08)	1.25 (0.97, 1.60)	1.00 (0.83, 1.22)
Adjusted^b^	0.92 (0.78, 1.10)	1.21 (0.93, 1.57)	1.00 (0.83, 1.21)
PM_2.5_ adjusted^d^	0.94 (0.77, 1.15)	1.15 (0.86, 1.55)	0.97 (0.79, 1.19)
PM_10_ adjusted^d^	0.96 (0.79, 1.15)	1.16 (0.87, 1.54)	0.99 (0.82, 1.20)
NO_2_ adjusted^d^	0.94 (0.78, 1.13)	1.17 (0.88, 1.56)	0.89 (0.63, 1.26)
O_3_ adjusted^d^	0.90 (0.74, 1.10)	1.10 (0.84, 1.45)	1.00 (0.82, 1.22)
BC adjusted^d^	0.90 (0.74, 1.10)	1.12 (0.84, 1.49)	0.99 (0.82, 1.22)

OR with 95% CI calculated per IQR increase in exposures.

a Reference category was normal birth weight 2500–4500 g.

b Adjusted for child’s sex, and maternal characteristics: age, smoking status, parity, obesity, and education level.

c Further adjusted for greenness exposure.

d Further adjusted for air pollutant exposures.

BC indicates black carbon; CI, confidence interval; HBW, high birth weight; LBW, low birth weight; NDVI, Normalized Difference Vegetation Index; OR, odds ratio; PM, particulate matter; PTB, preterm birth.

We found no evidence of effect modification by child sex (Table S3; http://links.lww.com/EE/A287).

Table S4; http://links.lww.com/EE/A287 shows the results of sensitivity analysis after excluding data from Tartu. We observed a similar association of the exposures with birth weight as in the main model with the strongest effect estimate change seen for NO_2_ (−52 g, 95% CI = −89, −14). In sensitivity analysis including either the presence of any maternal comorbidity or gestational age as potential mediators, we observed associations in the same direction but slightly higher in magnitude, especially for PM_2.5_ (Table S5; http://links.lww.com/EE/A287).

Overall, children with air pollution exposure above WHO recommendations had lower birth weight, compared with less exposed children. Specifically, PM_10_ exposure above 15 µg/m^3^ showed a stronger estimated association (−59 g, 95% CI = −109, −9) compared with PM_2.5_ above 5 µg/m^3^ (−27 g, 95% CI = −89, 34) and NO_2_ above 10 µg/m^3^ (−35g, 95% CI = −87, 18).

### Preconception exposure analysis

**Figure 4.**
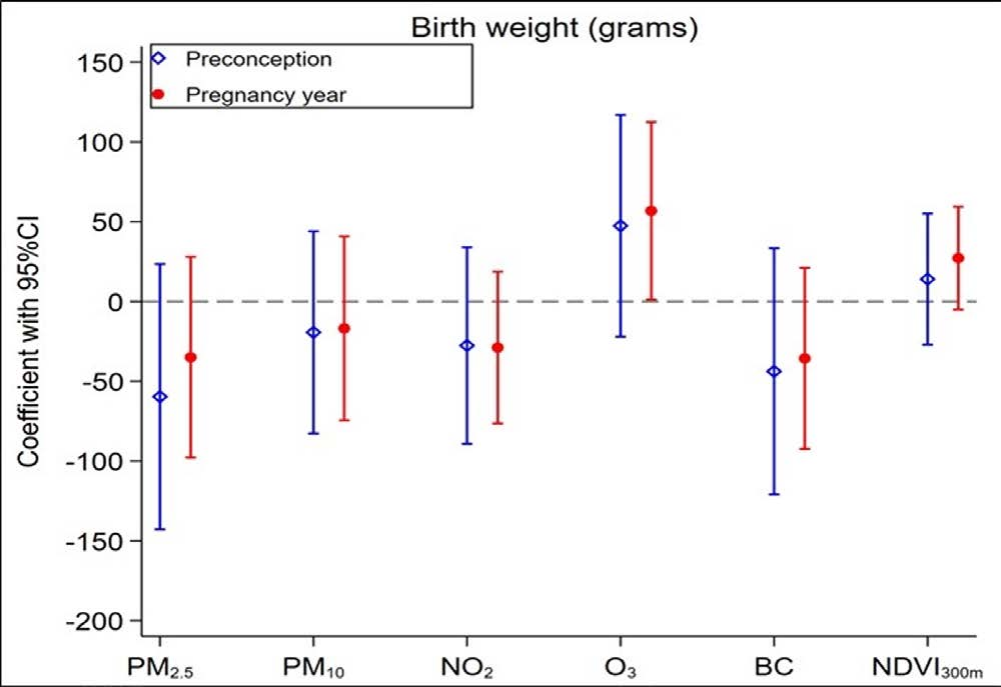
Estimated associations of residential exposures to air pollution and greenness over preconception and pregnancy year with birth weight in the subsample of children whose maternal addresses were assigned at RHINE II or III. Associations are expressed per IQR differences in air pollutants. All models were adjusted for child’s sex, and maternal characteristics: age, smoking status, parity, obesity, and education level. Preconception exposures were maternal exposures assigned at baseline (1990). BC indicates black carbon; CI, confidence interval; NDVI, Normalized Difference Vegetation Index; NO_2_, nitrogen dioxide; O_3_, ozone; OR, odds ratio; PM, particulate matter.

When restricting the analysis to the 2493 children having residential exposure assigned in 1990 (RHINE I) as a proxy of preconceptional exposures, the median (Q1–Q3) for pregnancy year was 1998 (1994–2001). We observed that the preconception and pregnancy year exposures were only low or moderately correlated, with Pearson correlations of 0.20 for NDVI_300m_ and between 0.50 and 0.60 for the air pollutants. Preconception and pregnancy year exposures showed similar estimated association with birth weight, which were consistent with the main analysis, although estimates were less precise (Figure 4).

## Discussion

Using data from a large sample of children born to women living in low to moderate air pollution countries in Northern Europe, we found that exposures to PM and NO_2_, both during pregnancy and in preconceptional periods, were associated with lower birth weight, whereas the opposite associations were seen for NDVI_300m_, an indicator of exposure to green spaces, and for ground-level O_3_. Neither air pollution nor greenness exposures were clearly associated with LBW, HBW, and PTB, probably due to the limited number of cases. The associations remained consistent across different adjustment sets, although they slightly shifted to the null in co-exposure analyses, that is, models including one air pollutant and NDVI_300m_. In utero exposure to PM_10_ above the AQG levels recommended by the WHO 2021 report was most strongly associated with decreased birth weight.

### Associations of air pollution and greenness with birth outcomes

The main findings of our study are consistent with previous studies, despite differences in exposure assessment, outcome definitions, and magnitude of the effect estimates. A systematic review reported pooled estimates of 27.6 g (95% CI = 48.5, 6.5) and 8.6 g (95% CI = 16.8, 0.5) decreased birth weight per 10 µg/m^3^ increase in PM_2.5_ and PM_10_, respectively.^16^ Similarly, a study reported a reduced birthweight of 19.9 g (95% CI = 18.6, 21.3) per IQR increase of 9.8 parts per billion in NO_2_ and 17 g (95% CI = 15.4, 18.6) per IQR increase of 0.14 µg/m^3^ in BC exposure during pregnancy.^42^ Our findings on O_3_ were opposite to those of other pollutants. Similar paradoxical effects of O_3_ on other health outcomes have been reported in other studies.^19,43,44^ Under the assumption that the association with NO_2_ is causal, this may be linked to the fact that concentrations of O_3_ are typically lower in urban areas where NO_2_ concentrations are high and higher in suburban and rural areas, where the emissions of nitrogen oxides from traffic are lower. The negative correlation between these pollutants is thought to be driven by the scavenging effect of nitrogen oxides on ground-level O_3_ through chemical reactions.^45^ Indeed, when conducting a post hoc analysis adjusted for both NO_2_ and O_3_ concentrations, the regression coefficient for O_3_ shifted to the null (11 g, 95% CI = −84, 105), while the association with NO_2_ remained consistent with the main analysis (−43 g, 95% CI = −128, 42), despite reduced precision in estimated associations due to multicollinearity.

We observed that the adjusted estimates for PM_2.5_, PM_10,_ and NO_2_ exposures with LBW were in the expected direction of adverse effects, as in previous studies^46^ and in line with the main analysis. The results on PTB may be chance findings due to small numbers of PTB events, as air pollution tends to be related to a higher risk of PTB rather than the opposite. This discrepancy could also be linked to heterogeneity of effects of environmental exposures on spontaneous vs. medically indicated PTB,^47^ a classification which was not available in our study.

Our results on greenness are consistent with previous studies. A study reported higher birth weight related to greenness exposure, with pooled estimates of 23.2 g (95% CI = 7.6, 38.7) for NDVI within 300 m buffer, and other buffer sizes showed similar strength of associations.^35^ Similarly, a European cohort study found that residential greenness exposure was associated with an increase in birth weight and lower odds for small gestational age. Further adjustment for NO_2_ and PM_2.5_ did not change the strength of the estimates.^48^

The secondary analysis of preconception exposure showed the potential long-term harm of air pollution. We observed a low correlation between exposures measured during pregnancy and preconception, which implies that these two indicators convey different information. Despite a smaller sample size, our findings suggest that preconception may be a susceptible period for birth outcomes and warrant further investigation.

### Plausible mechanisms

Air pollution and greenness may impact birth outcomes through various suggested biological mechanisms. PM may penetrate the maternal cardiorespiratory system, causing oxidative stress, blood coagulation, and placental dysfunction, reducing transplacental oxygen and nutrient exchange.^49,50^ PM_10_ induces inflammation that may impair placental perfusion.^51^ Similar mechanisms have also been described for NO_2_ and O_3_ as potent oxidants that trigger methemoglobin formation, leading to reduced oxygenation and potential hypoxia and hypoxemia in pregnant women.^52^

Recent evidence reports that BC particles can cross the placental barrier, impacting fetal development directly.^53^ Moreover, PM may accumulate in the maternal lung over a lifetime,^54^ potentially reaching the fetus during pregnancy through maternal-fetal exchange, even when inhaled preconceptionally.

Several plausible mechanisms have been explored to explain the beneficial effects of greenness exposure. It is suggested that greenness may directly remove air pollutants throughout its biological cycles.^55^ Additionally, air pollution sources may be lower in green spaces due to competitive land use with vegetation.^56^ Other suggested pathways include psychophysiological recovery, encouraging physical activity, and facilitating social cohesion.^55^

### Strengths and limitations

This study used data of adults that have been followed for 20 years and their offspring from seven Northern European cities. Through validated questionnaires, we collected comprehensive maternal and offspring individual-level information on several covariates related to our outcomes, thus enabling us to adjust for possible confounding. Maternal moving history was considered when assigning pregnancy year exposures. Moreover, we used a validated air pollution modeling system for high-resolution estimation of concentrations of air pollutants at maternal residential addresses over decades.

Despite the strengths, a few limitations warrant consideration. Although we considered moving history, our analysis assumed that the assigned exposures were valid for all the years the mothers lived at the residential addresses since we only had three time points of modeled exposure (1990, 2000, and 2010) and were not able to consider the variations in the exposures between the time points. We lacked the exact dates of birth, and therefore we used annual average exposures over the year of pregnancy as proxy indicators for 9-month pregnancy exposures. We acknowledge that this could be a source of exposure misclassification if the air pollution exposure levels during the 9 months of pregnancy differed substantially from the annual average concentrations. In addition, we considered residential exposure as a proxy for daily life exposure and did unfortunately not have data on work-life exposure or leisure outdoor exposures. Maternal reporting of residential history and pregnancy-related information may be subject to recall bias, moreover, the validation study covered only Norway.^22^ Using only NDVI as a greenness indicator, we further lacked information on the use of green spaces, accessibility, and types of vegetation that could be relevant for the proposed underlying mechanisms.^12^ We lacked data on annual temperature, potentially impacting our estimates.^57^ In addition, we did not have data on area-level socioeconomic status, and the possibility of residual confounding by other uncontrolled covariates cannot be excluded. Finally, preconception exposure windows varied across mothers, and we did not have a precise susceptibility window of the exposure.

## Conclusions

The findings of this study suggest that reducing air pollution exposure and increasing exposure to greenness, both before and during gestation, may contribute to healthier pregnancies, thereby improving the overall health of the next generations. Our results emphasize the need to adopt policies that target the reduction of air pollution emissions and exposure of the population, aimed at complying with WHO AQG limits. Ensuring sustainable urban planning to increase access of expectant women to green spaces may contribute to improve pregnancy outcomes.

## Conflicts of interest statement

The authors declare that they have no conflicts of interest with regards to the content of this report.

## Supplementary Material
