# Supplementary material for: Pregnancy outcomes as related to in utero exposure to air pollution and greenness: The Life-GAP Project
Source: Environ Epidemiol. 2024 Jun 21;8(4):e318. doi: 10.1097/EE9.0000000000000318 (PMC11196084; doi:10.1097/EE9.0000000000000318)
Supplement: Supplementary file 1 [file ee9-8-e318-s001.pdf]

Pregnancy outcomes as related to in utero exposure to air pollution and greenness: The Life-GAP Project

Supplementary Tables and Figures

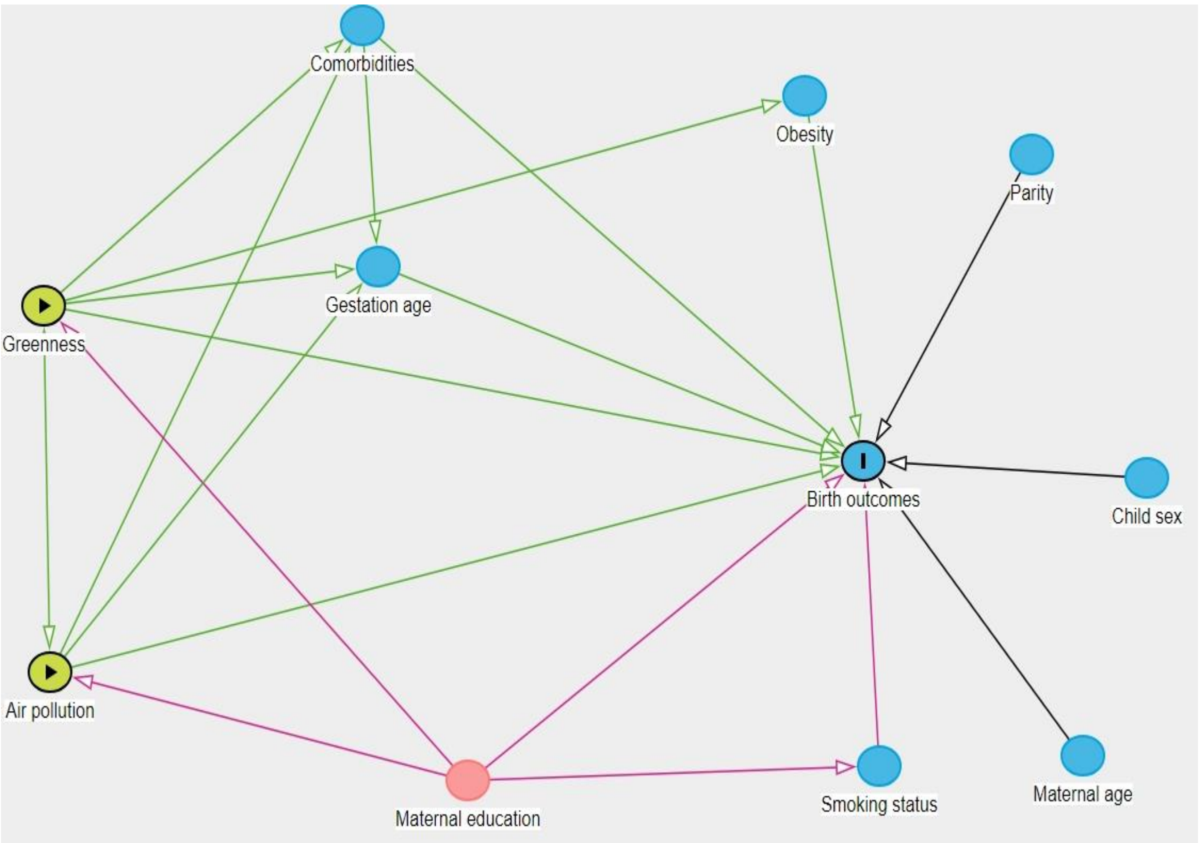

Figure S1. Directed Acyclic Graph (DAG) for the association of air pollution and greenness with birth outcomes.

Table S1. Descriptive statistics of LBW, HBW and PTB, by centre

Centre	n (%)	LBW n (%)	HBW n (%)	PTB n (%)
Aarhus	948 (22.1)	24 (2.7)	39 (4.3)	38 (4.0)
Bergen	751 (17.5)	26 (3.8)	20 (3.0)	55 (7.4)
Gothenburg	483 (11.3)	14 (3.2)	19 (4.3)	41 (8.6)
Reykjavik	494 (11.5)	16 (3.8)	40 (8.9)	51 (10.3)
Tartu	454 (10.6)	18 (4.2)	14 (3.3)	58 (13.2)
Umea	535 (12.5)	16 (3.4)	16 (3.4)	44 (8.5)
Uppsala	621 (14.5)	6 (1.1)	31 (5.5)	49 (8.1)

Abbreviations: HBW, high birth weight; LBW, low birth weight; PTB, preterm birth.

Table S2. Summary statistics of air pollutants and greenness exposures during pregnancy year.

Exposure	n	Mean (SD)	Percentile			IQR
			25th	50th	75th	
All centres						
$PM_{2.5}$ (μg/m^3)	4015	8.5 (3.90)	5.50	8.40	10.5	5.00
PM_{10} (μg/m^3)	4016	15.1 (6.30)	11.0	14.4	19.3	8.30
NO_2 (μg/m^3)	4015	14.6 (7.70)	9.10	14.0	20.1	11.0
O_3 (μg/m^3)	3665	54.9 (6.60)	49.5	54.7	59.7	10.2
BC (μg/m^3)	3665	0.48 (0.28)	0.27	0.47	0.68	0.41
$NDVI_{300m}$	3801	0.27 (0.23)	0.21	0.31	0.41	0.20

Abbreviations; SD, standard deviation; μg/m^3, micrograms per cubic meter ; $PM_{2.5}$, particulate matter with an aerodynamic diameter of ≤2.5μm; PM_{10}, particulate matter with an aerodynamic diameter of ≤10μm; NO_2, nitrogen dioxide; O_3, ozone; BC, Black Carbon; $NDVI_{300m}$, Normalized Difference Vegetation Index within 300-meter buffer.

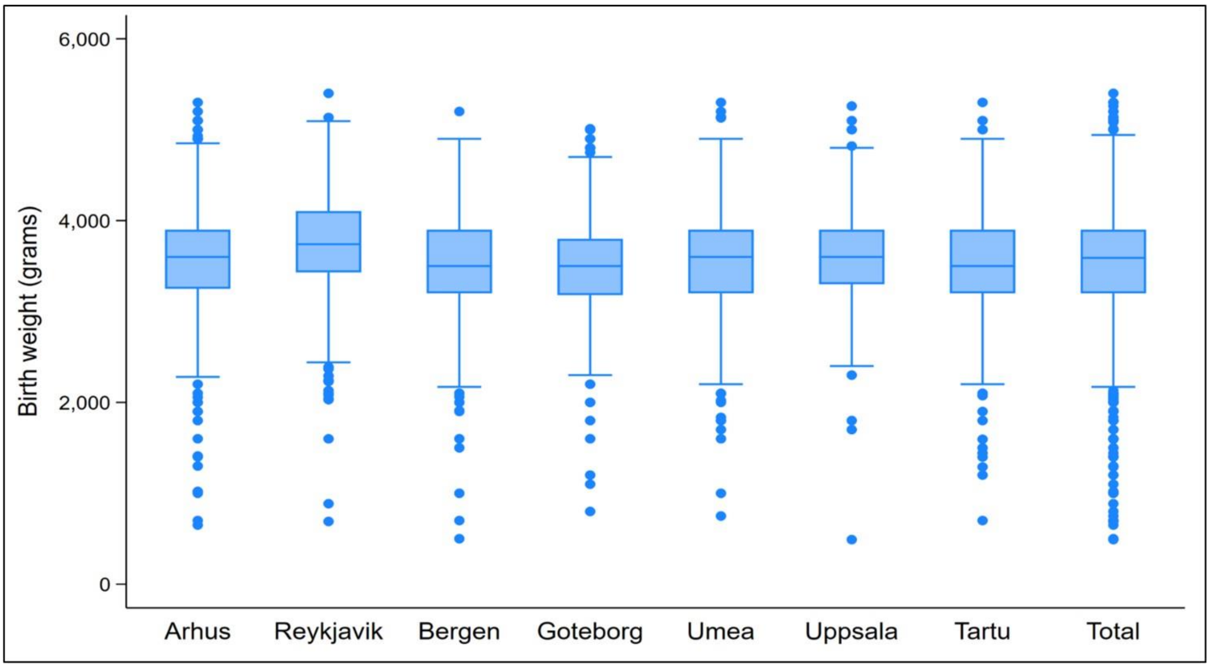

Figure S2. Distribution of birth weight by centre.

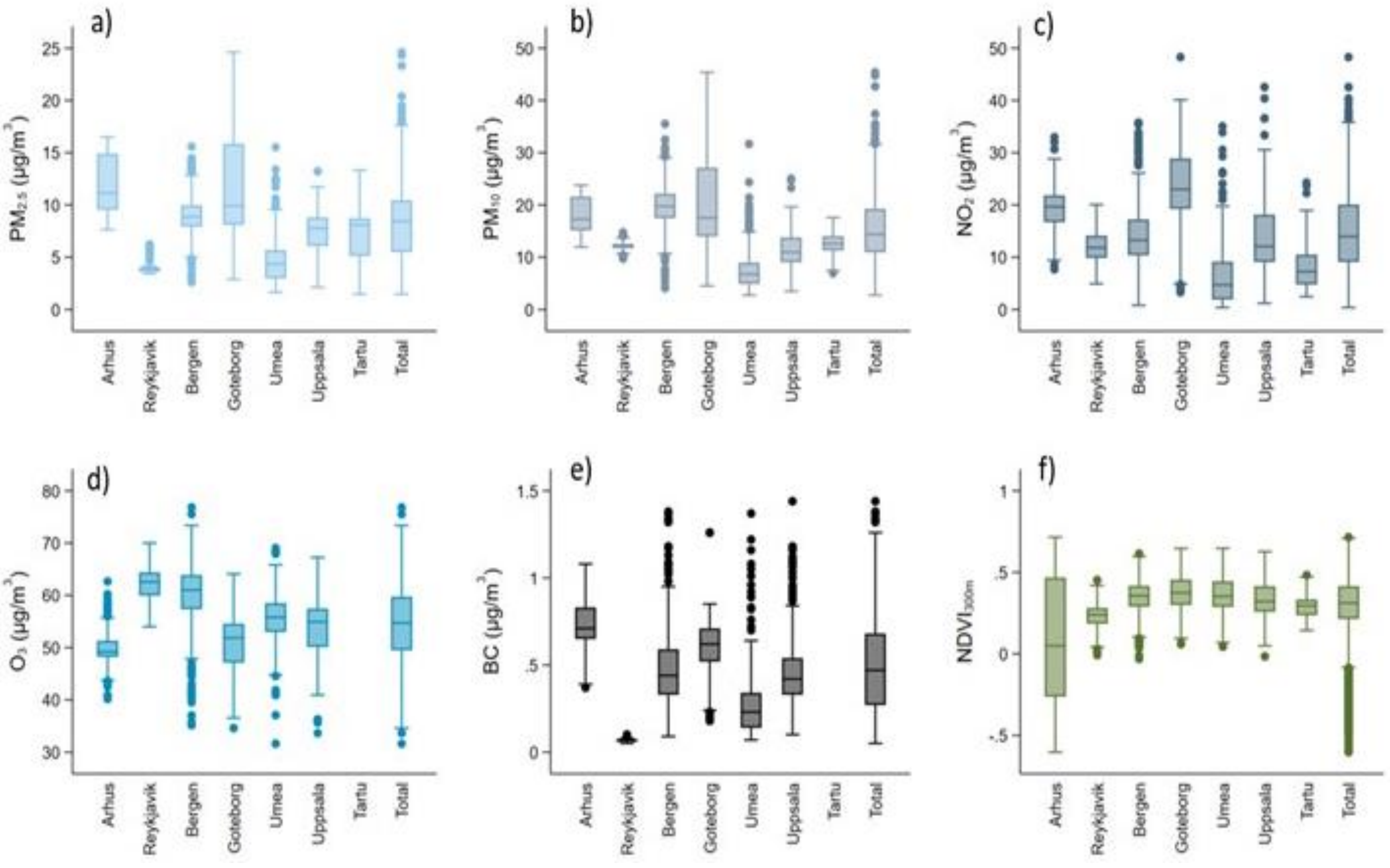

Figure S3**.** Distribution of residential exposure to air pollutants and greenness by centre; (a) $PM_{2.5}$, (b) PM_{10}, (c) NO_2, (d) O_3 , (e) BC and (f) NDVI

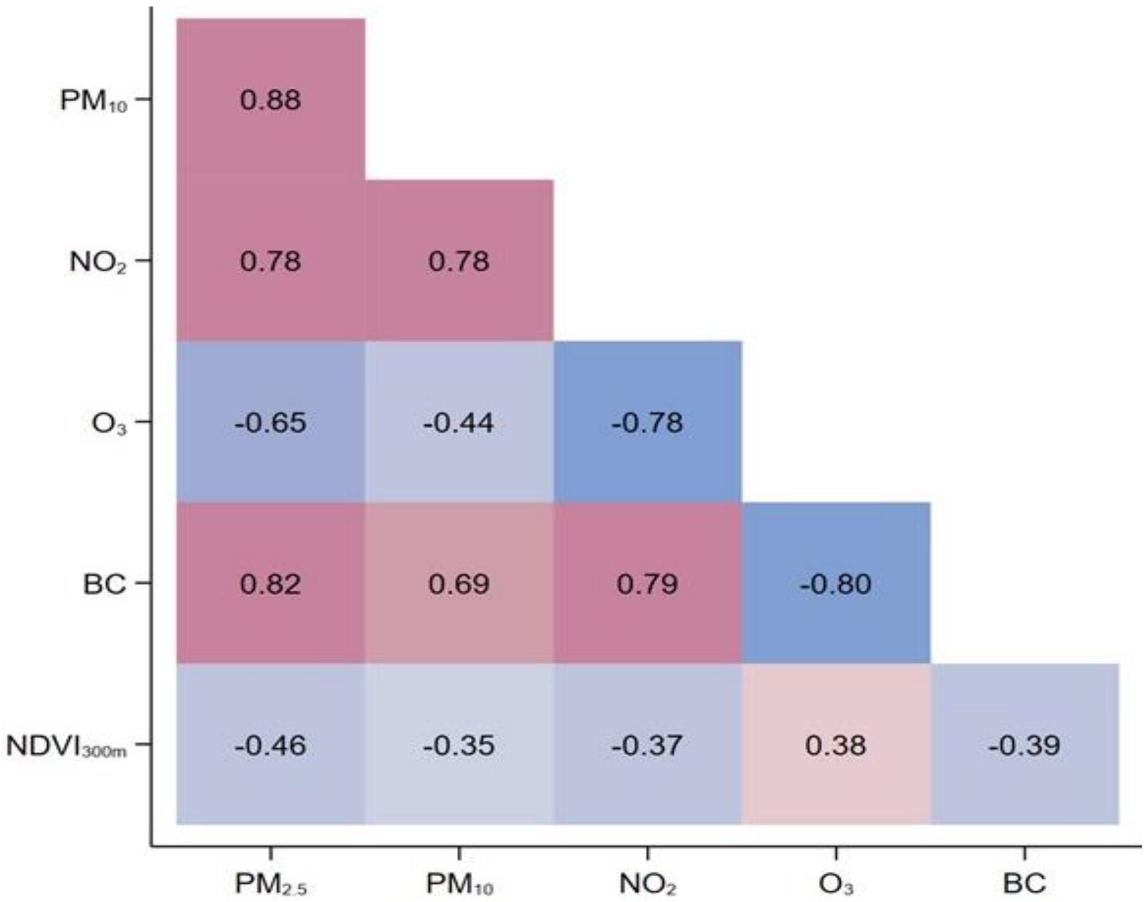

Figure S4. Pearson's r correlation coefficients for residential exposures to air pollutants and greenness during pregnancy year.$PM_{2.5}$, particulate matter with an aerodynamic diameter of ≤2.5µm; PM_{10}, particulate matter with an aerodynamic diameter of ≤10µm; NO_2, nitrogen dioxide; O_3, ozone; BC, Black Carbon; $NDVI_{300m}$, Normalized Difference Vegetation Index within 300-meter buffer. $PM_{2.5}$ (n =4016), PM_{10} (n=4016), NO_2 (n=4016), O_3 (n=3665), BC (n=3665), $NDVI_{300m}$ (n=3801)

Table S3. Estimated associations of residential exposures to air pollutants and greenness ($NDVI_{300m}$) during pregnancy and birth weight in grams stratified by child's sex.

Air pollutants	Child's sex		
	Male (n=1766) β (95% CI)	Female (n=1729) β (95% CI)	P value for interaction*
$PM_{2.5}$	-71(-113, -29)	-31 (-74, 11)	0.23
PM_{10}	-63 (-106, -19)	-33 (-76, 10)	0.33
NO_2	-54(-103, -6)	-32 (-78, 13)	0.23
O_3	62 (6, 118)	53 (0.65, 106)	0.49
BC	-59(-114, -3)	-35 (-91, 20)	0.49
$NDVI_{300m}$	21 (-5, 47)	19 (-6, 43)	0.90

Abbreviations: β, Coefficient ; CI, confidence interval; P value of the sex × exposure interaction terms from a joint model (males + females). β with (95% CI) calculated per IQR increase in exposures. All models are adjusted for maternal characteristics: age, smoking status, parity, obesity, and education level.*

Table S4. Estimated associations of residential exposures to air pollutants and greenness ($NDVI_{300m}$) during pregnancy and birth weight in grams. Sensitivity analysis excluding Tartu.

	All centres		Excluding Tartu	
Exposures	n	β (95% CI)	n	β (95% CI)
$PM_{2.5}$	3493	-49.46 (-82.84, -16.08)	3191	-56.72 (-91.75, -21.69)
PM_{10}	3493	-42.46 (-76.53, -8.38)	3191	-45.69 (-80.60, -10.78)
NO_2	3493	-43.04 (-79.30, -6.78)	3192	-51.69 (-89.51, -13.87)
$NDVI_{300m}$	3307	25.05 (6.55, 43.56)	3033	25.39 (6.59, 44.18)

Abbreviations: β, Coefficient ; CI, confidence interval.β with (95% CI) calculated per IQR increase in exposures. All models are adjusted for child's sex, and maternal characteristics: age, smoking status, parity, obesity, and education level.

Table S5. Estimated associations of residential exposures to air pollutants and greenness ($NDVI_{300m}$) during pregnancy and birth weight in grams. Analysis including potential mediators.

Exposures	Birth weight [a]<br>β (95% CI)	Birth weight [b]<br>β (95% CI)
$PM_{2.5}$	-62.94 (-94.55, -31.32)	-66.75 (-95.85, -37.64)
PM_{10}	-54.72 (-86.97, -22.47)	-54.81 (-84.74, -24.88)
NO_2	-43.77 (-78.83, -8.72)	-49.32 (-82.07, -16.58)
O_3	53.99 (13.52, 94.47)	52.16 (14.42, 89.91)
BC	-34.89 (-78.37, 8.58)	-35.08 (-76.05, 5.88)
$NDVI_{300m}$	35.89 (18.23, 53.55)	36.79 (20.61, 52.98)

Abbreviations: β, Coefficient ; CI, confidence interval. β with (95% CI) calculated per IQR increase in exposures. [a] Models including only presence of maternal comorbidities (at least one among gestational diabetes, hypertension, and proteinuria). [b] Models including gestational age (preterm, term, post term birth)

Table S6. Estimated associations of residential exposures to air pollution and greenness over preconception and pregnancy year with birth weight in the subsample of children whose maternal addresses were assigned at RHINE II or III.

	Preconception		Pregnancy year	
Exposures	n	β (95% CI)	n	β (95% CI)
$PM_{2.5}$	2092	-59.70 (-142.85, 23.47)	1970	-34.98 (-97.92, 27.93)
PM_{10}	2092	-19.36 (-82.70, 44.00)	1970	-16.85 (-74.51, 40.81)
NO_2	2091	-27.61 (-89.23, 34.00)	1969	-28.87 (-76.42, 18.68)
O_3	1842	47.46 (-22.05, 116.98)	1806	56.86 (1.23, 112.50)
BC	1842	-43.83 (-121.01, 33.35)	1806	-35.70 (-92.42, 21.03)
$NDVI_{300m}$	2075	14.01 (-27.11, 55.13)	1807	27.19 (-5.04, 59.43)

All models were adjusted for child's sex, and maternal characteristics: age, smoking status, parity, obesity, and education level. Preconception data was based on the assigned modelled air pollution and greenness maternal exposure at baseline (1990).
